# Supplementary material for: Differential Effects of Multiple Dimensions of Poverty on Child Behavioral Problems: Results from the A-CHILD Study
Source: Int J Environ Res Public Health. 2021 Nov 11;18(22):11821. doi: 10.3390/ijerph182211821 (PMC8624981; doi:10.3390/ijerph182211821)
Supplement: Supplementary file 1 [file ijerph-18-11821-s001.zip › ijerph-1456177-supplementary.pdf]

Supplemental Figure S1. Overlap of the three dimensions of poverty

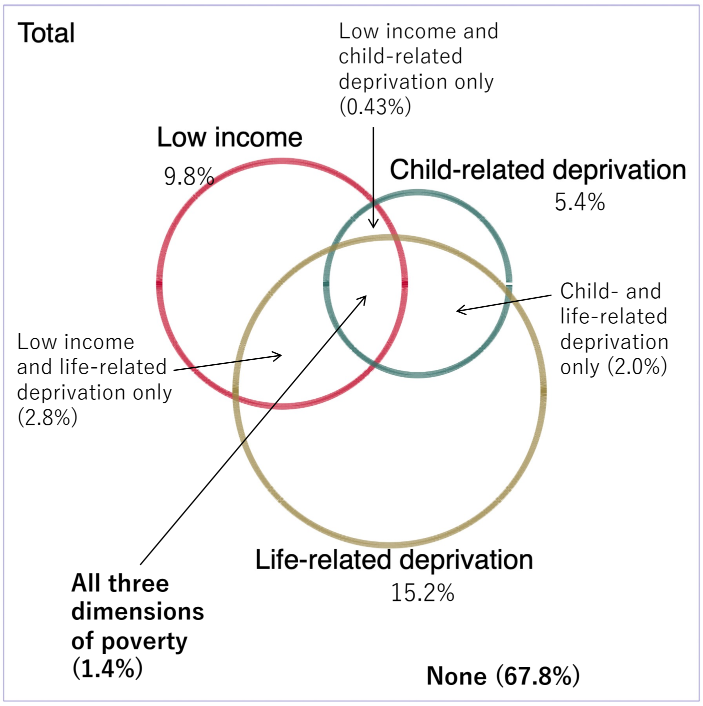

Supplemental Figure S2. The structural equation models

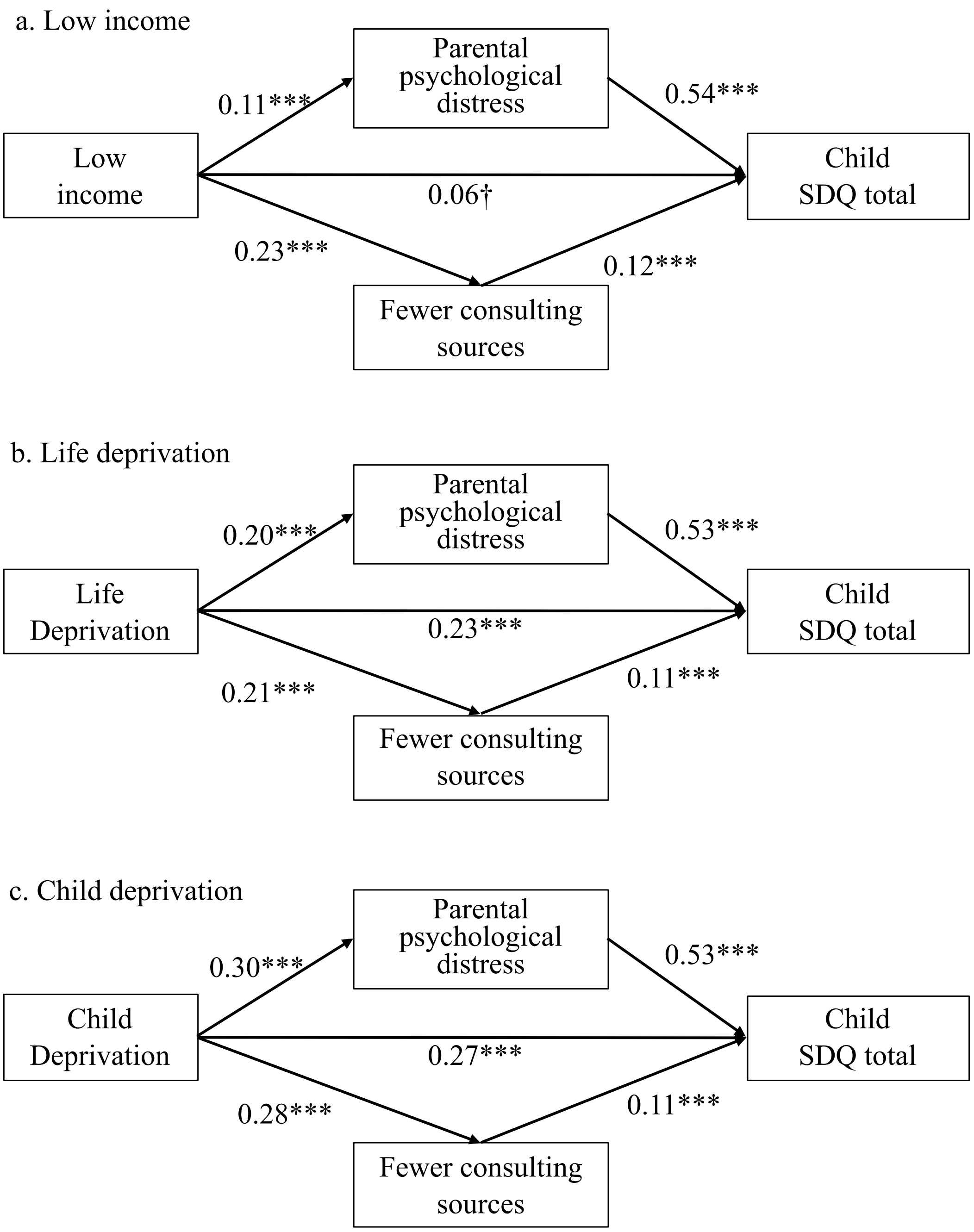

*** p<0.001, ** p<0.01, * p<0.05, † p<0.01
